# Monocytes/Macrophages play a pathogenic role in IL-23 mediated psoriasis-like skin inflammation

**DOI:** 10.1038/s41598-019-41655-7

**Published:** 2019-03-29

**Authors:** Yibing Wang, Rebecca Edelmayer, Joe Wetter, Katherine Salte, Donna Gauvin, Laura Leys, Stephanie Paulsboe, Zhi Su, Isaac Weinberg, Marian Namovic, Stephen B. Gauld, Prisca Honore, Victoria E. Scott, Steve McGaraughty

**Affiliations:** Dermatology, AbbVie Inc., 1 North Waukegan Rd., North Chicago, IL 60064 USA

## Abstract

Psoriasis is an immune-mediated inflammatory skin disease that affects millions worldwide. Studying immune cells involved in psoriasis pathogenesis is essential to identify effective and safe therapeutics for the disease. Using human psoriasis skin, activated macrophages were observed in both lesional and non-lesional skin, but were elevated in lesional skin. Activation of the IL-23/IL-17 pathway is integral to the development of psoriasis. To further characterize the monocyte/macrophage (Mon/Mac) population when the IL-23 pathway is activated, a murine model of intradermal injection of IL-23 was used. Flow cytometry revealed that Mon/Mac cells were the dominant immune population, particularly late in the model, highlighted by strong presence of Ly6C^hi^MHC II^hi^ cells. The Mon/Mac cells were also shown to have high expression for TNFα but not IL-17A. Prophylactic dosing of a CSF-1R inhibitor to deplete Mon/Mac cells significantly reduced several inflammatory mediators from the skin tissue suggesting a pathogenic role for Mon/Mac. Treatment dosing of the inhibitor produced a less robust effect. Mon/Mac cells were also differentiated by levels of Ki67 and TNFα expression. These data point to an important contribution of Mon/Mac cells in IL-23 related skin inflammation and suggest that these cells are a significant player in the underlying pathophysiology of psoriasis.

## Introduction

Psoriasis (Ps) is a chronic auto inflammatory skin disease that affects 1 to 2 percent of the U.S. population and 0.2 to 4.8 percent of the population world-wide^1–3^. The most common clinical variant of Ps is Psoriasis vulgaris (a.k.a. Plaque Psoriasis) affecting approximately 85 to 90 percent of diagnosed Ps patients^2^. Histopathologically, Ps vulgaris manifests with four distinctive features: thickening of epidermis, elongated rete ridges, parakeratosis, and infiltration of diverse types of leukocytes in both the epidermis and dermis. The leukocyte infiltration includes cells from both the innate and adaptive immune systems that reflect a complex interplay of the two immune systems during disease progression. A critical role for T cell subsets in the pathogenesis of Ps has been well established and reviewed by others^3,4^. In contrast, a defined role for myeloid lineage cells, monocytes and macrophages in particular, has continued to evolve. Previous studies linked macrophage involvement in Ps to its production of TNFα^5^ and also identified a subpopulation of CD163^+^ macrophages that were classically activated in Ps lesional skin^6^. Different preclinical models of Ps also have supported a role for macrophages in the pathogenesis of the disease^7–10^.

IL-23 is an IL-12 cytokine family member composed of two subunits, p19 and p40. Human genetic association studies have revealed a strong linkage between IL-23R and Ps^11–13^. IL-12 and IL-23 have independent roles in Ps^14–16^. Confirmation of the importance of the IL-23 pathway in the pathogenesis of Ps follows with the recent development of successful therapeutics that modulate this pathway^17^. Thus, murine models that activate the IL-23/IL-17 axis in skin have been developed to further interrogate the mechanistic consequences associated with stimulation of this pathway^18–21^. Indeed, a model using intradermal injection of IL-23 in mice has demonstrated a relatively strong transcriptional match to human Ps when compared to other murine models^20^. Similar IL-23 murine models have shown marked infiltration of T cells followed by robust accumulation of macrophages^18^ and also a role for monocyte derived dendritic cells (moDCs) in the observed inflammation^19^.

In this study, the role of monocyte/macrophage (Mon/Mac) was further examined in relation to Ps and IL-23 related inflammation. In human lesional skin, activated macrophages increased with a unique distribution pattern by congregating in sites of active inflammation. Using the IL-23 intradermal injection model, a robust accumulation of Ly6C^hi^MHCII^lo/hi^ Mon/Mac cells contributed to disease progression. Pharmacological depletion of the Mon/Mac population both prior to and after established inflammation significantly reduced the disease phenotype. Additionally, it was found that Mon/Mac cells were the main source of TNFα and they regulated IL-17A production during disease progression. Thus, the current data adds to the understanding of how Mon/Mac cells contribute to the pathogenesis of Ps and in particular their role following activation of the IL-23/IL-17 pathway.

## Results

### Activated macrophages were increased in lesional human psoriasis skin compared to non-lesional skin

**Figure 1:**
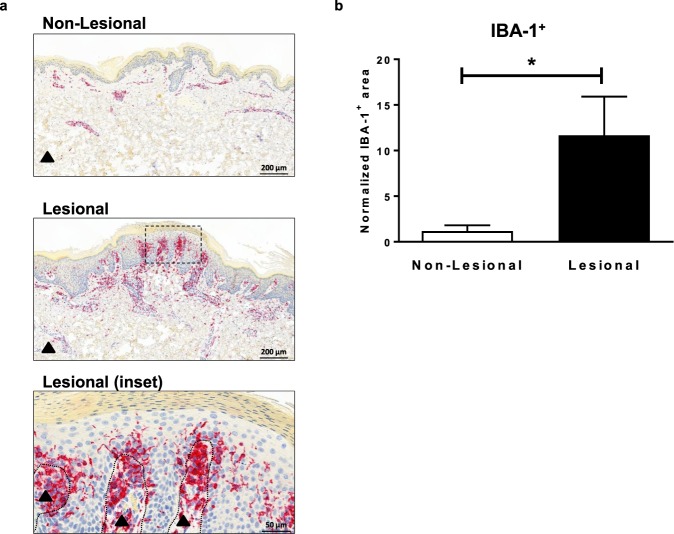
IBA-1^+^ macrophages were increased in lesional human Ps skin. IBA-1 IHC staining of non-lesional and lesional skin of the Ps patients (n = 4) (**a**) representative images of IHC staining of IBA-1 (red) in epidermis and dermis (black filled triangle) area. Inset image represents active parakeratosis area within the lesional skin biopsy. IBA-1^+^ macrophages resided in both epidermis and dermis area and congregated under the active parakeratosis region. Section of whole skin biopsies of both lesional and non-lesional were scanned with digital scanner at 20X objective. The representative images within the skin section were from 8X digital magnification. Inset area of the lesional skin was from 32X digital magnification. (**b**) quantification of IBA-1^+^ signal of non-lesional and lesional skin of Ps patients. IBA1^+^ staining was normalized to the length of biopsy epidermis analyzed for each donor and presented as area (μm^2^) divided by length (μm). Data are shown as mean ± SEM.; *P < 0.05.

Macrophages were reported to be abundant in Ps skin compared to normal skin^6^. In order to confirm the observation and to further evaluate the levels and distribution of macrophages in Ps patient skin, immunohistochemistry was performed using IBA-1^22,23^ to identify activated macrophages. The level of IBA-1^+^ signal was significantly elevated in Ps lesional skin compared to non-lesional skin taken from the same patient (Fig. 1). IBA-1^+^ macrophages were present in both dermal and epidermal layers of skin. The most robust signal was within the dermis beneath the parakeratotic area, suggesting that macrophages were recruited to the site of active inflammation within the lesional plaque.

### CD64^+^ Mon/Mac dominated the increase of immune cells following IL-23 induced skin inflammation

**Figure 2:**
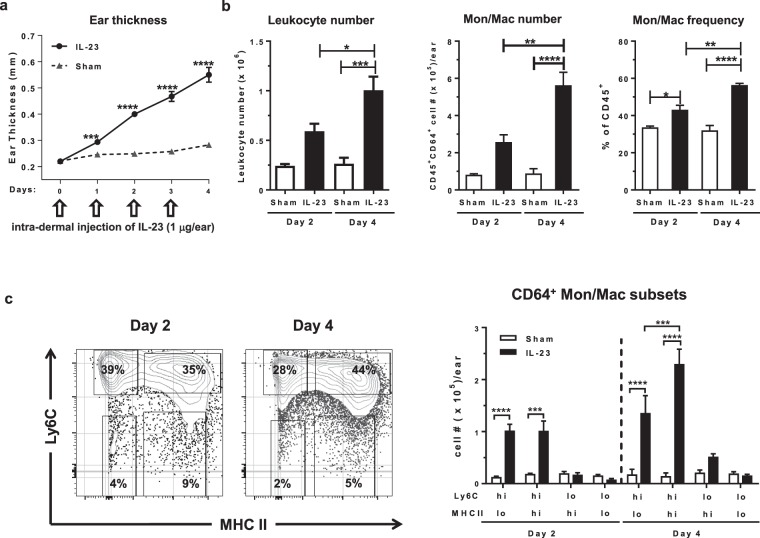
CD64^+^ Mon/Mac population dominated the increase of immune cells in the murine model of IL-23 induced skin inflammation. (**a**) Daily ear thickness measurement in IL-23 and PBS + 0.1% BSA (i.e. sham) injected mice (n = 6–8). (**b**) Enumeration of ear leukocytes (CD45^+^ live cells, left) and ear Mon/Mac (CD45^+^CD64^+^ live cells, center) and frequency of ear Mon/Mac (CD45^+^CD64^+^ live cells, right) on day 2 and day 4 of IL-23 and sham injected mice (n = 4). All values were determined by flow cytometry. (**c**) Representative 2-dimentional couture plot (left) of cell surface expression of Ly6C and MHC II on Mon/Mac cells from IL-23 injected ears at day 2 and 4 determined by flow cytometry and respective enumeration (right) of ear Mon/Mac subsets (CD45^+^CD64^+^) based on Ly6C/MHC II expression levels on days 2 and 4 of IL-23 and sham injected mice (n = 4). Flow cytometry gating strategy is detailed in Supplemental Fig. S2. Numerical data are shown as mean ± SEM.; *P < 0.05; **P < 0.01; ***P < 0.001; ****P < 0.0001.

In a previous study, we observed an increase of Mon/Mac cells in a murine model of IL-23 induced skin inflammation^18^. To delineate the role of Mon/Mac population in the pathogenesis of this inflammation, we further characterized these cells in this model (Fig. 2). Daily intradermal injections of IL-23 induced a robust ear inflammation represented by significantly increased ear thickness up to the end of the study (i.e. day 4, Fig. 2a), and by elevated leukocyte infiltration compared to sham injected animals (Fig. 2b). CD64, a high affinity IgG receptor FcγRI^24^, was used to discriminate between dendritic cells (DC) and Mon/Mac populations. On day 2 of the IL-23 model, 43% (P < 0.05) of the infiltrated leukocytes were CD64^+^ Mon/Mac and this continued to rise to 56% (P < 0.001) by day 4 (Fig. 2b). Thus, as the disease progressed, the accumulation of Mon/Mac cells predominated the immune cell influx. This suggests that Mon/Mac may be involved in the maintenance and/or exacerbation of the observed ear inflammation.

To further discriminate the CD64^+^ Mon/Mac population in the IL-23 injected skin, the expression levels of Ly6C/MHC II was analyzed (Fig. 2c, Supplementary Fig. S2). Blood monocytes that are Ly6C^hi^ (classical monocytes) have been reported to be recruited into inflamed tissue and are precursors for mononuclear phagocytes^25^. Differential expression levels of Ly6C and MHC II can facilitate further discrimination of Mon/Mac subsets in skin^26^. Four sub-populations were identified in the skin of IL-23 injected mice: Ly6C^hi^MHC II^lo^, Ly6C^hi^MHC II^hi^, Ly6C^lo^MHC II^hi^, and Ly6C^lo^MHC II^lo^. Both Ly6C^hi^MHC II^lo^ and Ly6C^hi^MHC II^hi^ cells were significantly increased (P < 0.001) over the course of the IL-23 model. The increase of these two populations was equally dominant on day 2, 44% and 45% respectively (Fig. 2c, Supplementary Fig. S1). However on day 4, the Mon/Mac compartment was dominated by Ly6C^hi^MHC II^hi^ cells (53% of Ly6C^hi^MHC II^hi^ vs. 30% of Ly6C^hi^MHC II^lo^, P < 0.001). Although both of these two Ly6C^hi^ Mon/Mac subsets were elevated on day 2, the higher proportion of Ly6C^hi^MHC II^hi^ cells within the CD64^+^ Mon/Mac compartment on day 4 suggests that these cells may be derived from the Ly6C^hi^MHC II^lo^ monocytes and accumulated progressively in the tissue. A similar observation has been reported in the inflamed gut^27^. This is consistent with the view that a dynamic Ly6C^hi^ monocyte population exists in inflamed tissue^28^.

### Mon/Mac is a major contributor to IL-23 model pathogenesis

**Figure 3:**
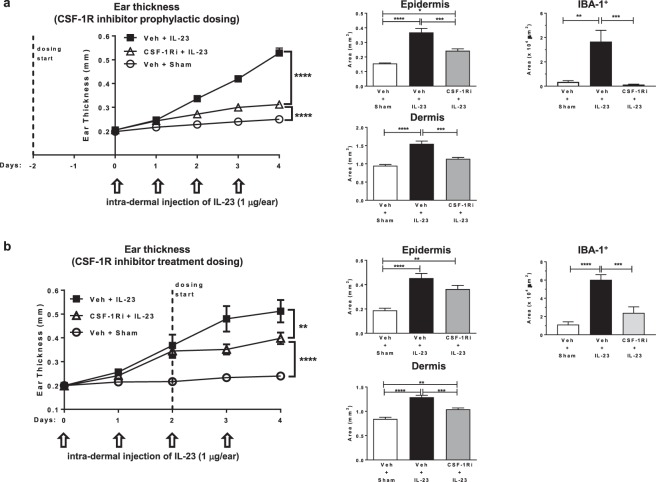
Both prophylactic and treatment dosing of small molecule CSF-1R inhibitor (JNJ-40346527) reduced IL-23 induced skin pathophysiology. (**a**) Prophylactic and (**b**) treatment dosing of JNJ-40346527 (CSF-1Ri + IL-23) or vehicle (Veh + IL-23) in IL-23 injected mice. Sham injection controls dosed with vehicle (Veh + Sham) were also included in both studies. Daily ear thickness measurement (left), epidermal and dermal area measurements (center), and IBA-1^+^ staining (right) are shown for both studies (n = 6–8). For each type of staining, one 5 mm length of ear sample section was analyzed per animal. Staining was reported as tissue area (mm^2^) for H&E and tissue staining area (µm^2^) for IBA-1. Data are shown as mean ± SEM. **P < 0.01; ***P < 0.001; ****P < 0.0001.

**Figure 4:**
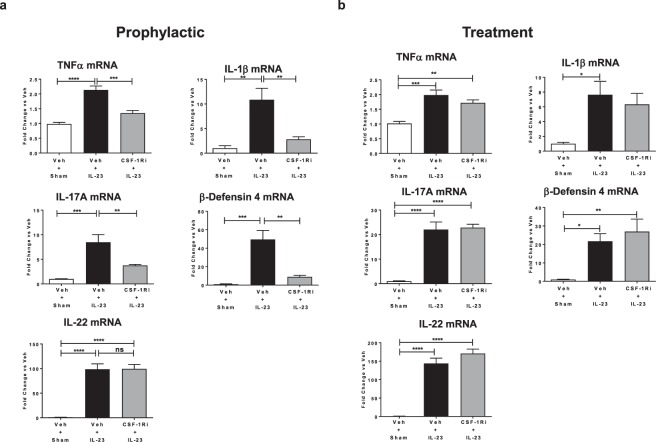
JNJ-40346527 modulated IL-23 induced proinflammatory mediators in ear skin. (**a**) Prophylactic dosing of JNJ-40346527 reduced the mRNA expression levels of TNFα, IL-1β, IL-17A, and β-defensin 4 but not IL-22 that were all elevated by IL-23 injections. (**b**) Treatment dosing of JNJ-40346527 did not significantly alter the levels of these genes, but there was a trend for reduction in TNFα, and IL-1β levels. Sham injection controls dosed with vehicle were also included in both studies. Data calculated as fold change compared to sham injected vehicle dosing group. Data are shown as mean ± SEM. *P < 0.05; **P < 0.01; ***P < 0.001; ****P < 0.0001.

Generation, proliferation, and maintenance of the Mon/Mac population depend on signals mediated through the CSF-1R protein^29^. To assess the pathophysiological role of Mon/Mac in the IL-23 model, JNJ-40346527, a selective oral CSF-1R tyrosine kinase inhibitor^30,31^ was administered to deplete the Mon/Mac population. Animals dosed with the inhibitor prior to the first injection of IL-23 displayed a significant decrease in ear swelling (78%, P < 0.0001) as well as epidermal (59%, P < 0.001) and dermal areas (68%, P < 0.0001) compared to vehicle dosed animals on day 4 (Fig. 3a). IBA-1 immunohistochemistry confirmed complete depletion of macrophages (P < 0.001) following this prophylactic administration of JNJ-40346527. Additionally, the mRNA expression of pro-inflammatory genes, TNFα, IL-17A, IL-1β and β-defensin 4 in the ear skin was decreased in a range of 63–84% (P < 0.01) after JNJ-40346527 administration (Fig. 4a). Interestingly, gene transcripts of IL-22, a T cell cytokine that has been reported to drive epidermal hyperplasia and keratinocyte proliferation^32^, was significantly elevated (P < 0.0001) after IL-23 exposure but was not affected by the JNJ-40346527 induced Mon/Mac depletion.

In the next experiment, JNJ-40346527 was delivered after inflammation was established in the IL-23 model to determine effects of attenuating Mon/Mac levels at this stage of the disease. Thus, JNJ-40346527 was administered on day 2 (Fig. 3b) when there was already a significant rise (Fig. 2a, P < 0.0001) in ear thickness. Except for mRNA expression, treatment dosing of JNJ-40346527 showed similar trends to the prophylactic dosing but the degree of effect was more modest (Fig. 3b). IBA-1 staining of skin samples from day 4 confirmed a partial depletion of macrophages in dosed animals (P < 0.001), and correspondingly JNJ-40346527 reduced ear swelling by only 42% (P < 0.01), as well as epidermal and dermal area by 34% (non-significant) and 55% (P < 0.001), respectively. This partial reduction of inflammation was also accompanied by weak non-significant decreases of TNFα (28%) and IL-1β (20%), but no changes in mRNA expression for IL-17A, or β-defensin 4 (Fig. 4b), which were decreased with prophylactic dosing. Taken together, these data strongly suggest that Mon/Mac cells play a significant role during the pathogenesis and progression of IL-23 mediated skin inflammation.

### Mon/Mac is the major immune contributor for TNFα production in IL-23 mediated skin inflammation

**Figure 5:**
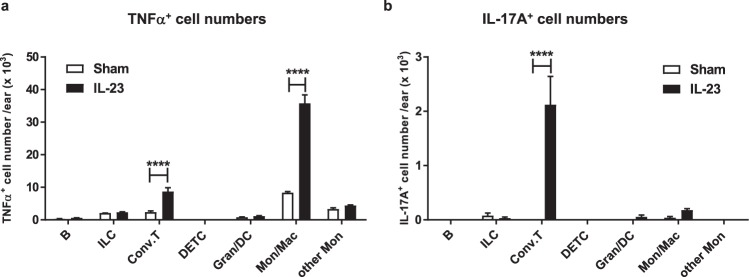
Mon/Mac and T cells were the predominant immune cells that produced TNFα and IL-17A, respectively. Number of (**a**) TNFα^+^ cells and (**b**) IL-17A^+^ cells in the ears of sham and IL-23 injected mice at day 4 (n = 6–8). The cellular lineage, TNFα and IL-17A expression were all determined by flow cytometry (detailed gating hierarchy in supplemental materials). Data are shown as mean ± SEM. ****P < 0.0001. B, B cells; ILC, innate lymphocytes; Conv.T, conventional T cells (i.e. αβ T cells and dermal γδ T cells); DETC, Dendritic epidermal T cells; Gran/DC, granulocytes and dendritic cells; Mon/Mac, monocytes/macrophages; other Mon, CD11b^lo^CD64^−^ monocytes.

TNFα and IL-17A are two inflammatory cytokines that have been clinically proven to be pathogenic for Ps^33,34^. Our group has shown that neutralizing either TNFα or IL-17A with antibodies at clinically relevant doses was efficacious in the IL-23 model^18^ and the current study demonstrated that both of these cytokines were elevated in the model as well. However, these cytokines were differentially modulated by administration of JNJ-40346527 (Fig. 4). In order to place these results in context, intracellular protein staining was performed using flow cytometry to identify immune cells that were TNFα or IL-17A positive from skin samples taken on day 4 of the IL-23 model (Fig. 5 and Supplementary Fig. S4). The TNFα^+^ leukocytes were predominantly from the Mon/Mac population and to a lesser extent from conventional T cells. The IL-17A^+^ leukocytes were primarily localized to the T cell population, in contrast to Mon/Mac, as well as neutrophils which were IL-17A negative and very few of them were TNFα positive (Fig. 5 and Supplementary Fig. S4). These data suggests that Mon/Mac may contribute to disease pathogenesis in the IL-23 model by directly producing TNFα and by indirectly regulating IL-17A levels from T cells.

**Figure 6:**
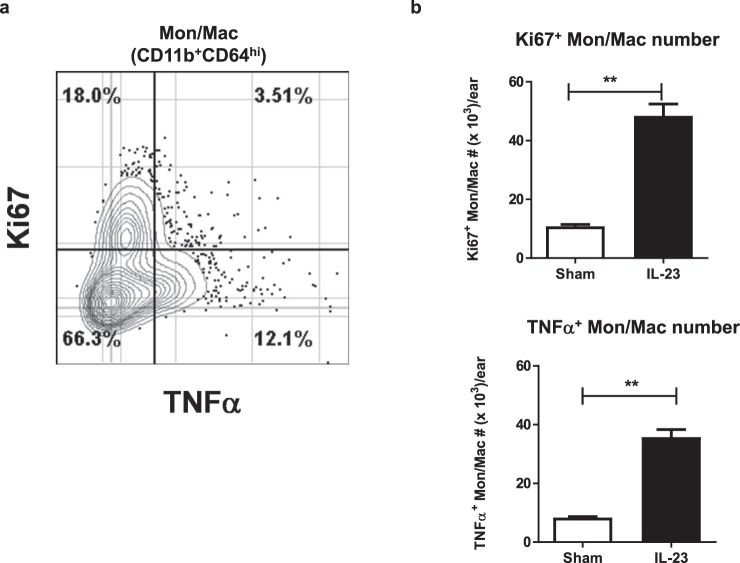
Two distinct Mon/Mac (Ki67^+^ and TNFα^+^) subsets increased in IL-23 inflamed skin. (**a**) Representative 2-dimentional couture plot of TNFα and Ki67 expression in Mon/Mac cells from IL-23 injected ears at day 4 determined by flow cytometry. The quad gate that separates TNFα and Ki67 positive/negative was determined based on comparing fluorescent staining from IL-23 ears to those from naive ears and IL-23 injected ears without the specific fluorescent antibody. Numbers indicate the frequencies of the cell population within the quad gates. (**b**) Enumeration of Ki67^+^ and TNFα^+^ Mon/Mac cells in ears of sham and IL-23 injected animals (n = 6–8). Frequencies of Ki67^+^ and TNFα^+^ Mon/Mac cells were determined as described in (**a**) and were used to calculate the number of cells per ear. Data are shown as mean ± SEM. **P < 0.01.

It was noteworthy that in addition to a TNFα^+^ Mon/Mac population in the IL-23 model, a separate population that was Ki67^+^ was also recognized (Fig. 6a). Ki67 is a well-known cellular proliferative marker^35^. Like the TNFα^+^ Mon/Mac population, Ki67^+^ Mon/Mac population was also significantly elevated (P < 0.01) by day 4 in the IL-23 model (Fig. 6b). The ratio of Ki67^+^ Mon/Mac to TNFα^+^ Mon/Mac in the Day 4 diseased ear was 1.35 indicating the presence of more of Ki67^+^ cells than TNFα^+^ cells. Thus, there are two functionally distinct Mon/Mac populations that increase following activation of the IL-23 pathway. One population is proliferative and the other is differentiated towards producing TNFα.

## Discussion

Ps is an auto-inflammatory skin disease with a complex pathophysiology that includes diverse cell types and cytokine pathways^4^. Within this complex biology, T cells are the most studied immune cell population with multiple subsets linked to the development of skin lesions^36–39^. Meanwhile, understanding the role of innate immune cells in Ps pathogenesis, in particular macrophages, is still evolving. Consistent with a previous report^6^, macrophage levels in the current study were increased in the lesional skin of human Ps patients relative to adjacent non-lesional skin from the same patient. These macrophages were localized to both dermal and epidermal areas of the lesional skin and tended to congregate beneath the parakeratotic areas in the diseased skin. This implies that macrophages were recruited to a site of active inflammation within the lesional skin, and supports the idea that macrophages are important for the pathogenesis of the disease, as reported by others using preclinical models^7–10^.

Recent genetic and clinical data have demonstrated that the IL-23/IL-17 pathway is a key driver in human Ps^17^. Thus, animal models that activate this axis have utility to study its mechanistic undertones and understand possible contributions to Ps. Intradermal injection of IL-23 in rodents, which has been used by several groups including ours^18–21^, has been reported to be a mechanistically useful model, and when compared to other murine models, better resembles human Ps at a transcriptomic level^20^. Using a four-day IL-23 injection model, the current study showed that the Mon/Mac population was a significant component of the infiltrating leukocytes in the inflamed skin and was the predominant cell type by the final day of the model. Four subsets of the Mon/Mac population were identified based on the detection of high levels of surface CD64 expression and then sub-divided into groups with high or low levels of surface Ly6C and MHC II molecules. Two of the subsets with high expression of Ly6C (Ly6C^hi^) were similarly elevated by day 2 and continued to increase up to at least day 4, but the Ly6C^hi^ MHC II^hi^ cells increased to a greater degree on day 4 than the Ly6C^hi^ MHC II^lo^ cells.

The tissue Mon/Mac cells in the current study were also found to be the prevalent immune cell population expressing TNFα indicating a proinflammatory contribution. The proinflammatory role was further substantiated by significant attenuation of several related disease endpoints following depletion/reduction of the Mon/Mac population in the IL-23 model. The apparent proinflammatory/pathogenic role of Mon/Mac in the current study is similar to the proposed role of moDCs in a previous report using an IL-23 injection model^19^. However, the majority of the Mon/Mac cells in the current study did not fall under the moDC lineage since they were high in CD64 expression, a marker that Singh’s group employed to differentiate moDCs from macrophages. Thus, monocyte derived cells other than moDCs, in particular Ly6C/MHC II expressing macrophages, can also be recruited following activation of the IL-23 pathway and contributed to the psoriasis-like skin inflammation.

The dynamic change towards a predominant Ly6C/MHC II Mon/Mac subset over time suggests that like moDCs, these Mon/Mac cells can mature during the progression of inflammation. The Ly6C^hi^MHC II^lo^ cells may be monocyte precursors in blood that enter tissue^27^ and are still highly proliferative which may have been reflected by the high levels of Ki67^+^ cells. These cells may then give rise to the MHC II “high” cells, which would be fully differentiated macrophages that no longer proliferate but produce inflammatory cytokines such as TNFα. Thus, it appears that IL-23 induced skin inflammation is partially a consequence of monocytes that were recruited to the tissue and then further differentiated into subsets that contributed to the disease pathophysiology. Since this observation was induced by IL-23 injection, this finding may be generalizable and possibly translatable to diseases like Ps that are linked to IL-23 pathway. The diversity of monocyte derived subsets may reflect a heightened cellular plasticity of monocytes during inflammation^28^. A deeper characterization of the Mon/Mac/DC subsets in the inflamed skin tissue is needed to further elucidate the individual and possibly redundant roles.

A selective CSF-1R tyrosine kinase inhibitor, JNJ-40346527, was used to deplete/reduce Mon/Mac levels in the IL-23 model^31^. CSF-1R is a hematopoietic growth factor receptor for CSF-1 and IL-34 that specifically regulates the homeostasis and development of mononuclear phagocytes, particularly monocytes and macrophages^25,29^. Both prophylactic and treatment dosing of JNJ-40346527 significantly reduced the IBA1^+^ macrophages and subsequently ear swelling in IL-23 injected mice. These effects on ear thickness, particularly with prophylactic dosing, were accompanied by reduction of TNFα mRNA expression. The effect on TNFα appears likely to be a direct consequence of reducing the Mon/Mac population as these cells were shown to be TNFα positive. Prophylactic dosing of JNJ-40346527 also significantly reduced IL-17A mRNA expression. The effect on IL-17A appears to be a downstream consequence of decreasing the Mon/Mac cells as it was demonstrated that T cells, but not the Mon/Mac cells, were the major class expressing IL-17A. Meanwhile, administration of JNJ-40346527 after establishing inflammation significantly reduced ear swelling but did not affect IL-17A expression. This suggests that there is a need for substantial reduction in Mon/Mac levels (and probably TNFα expression) to modulate IL-17A. Thus, treatment with JNJ-40346527 at a more advanced stage of the disease is likely not sufficient to totally overcome the “inflammatory momentum” resulting in partial efficacy. In contrast to the regulation of IL-17A, IL-22 expression was unaffected, suggesting a separate feedback loop regulating IL-22 production and possibly a different cellular subset (i.e., Th22) not affected by Mon/Mac reduction. An alternative approach to reduce Mon/Mac levels would have been to use clodronate liposomes which act intracellularly to induce apoptosis in phagocytic cells^40^. Administration of clodronate liposomes was efficacious in different mouse models of Ps-like inflammation^7,8,10^. Both approaches can functionally reach the same goal of reducing Mon/Mac levels, but ease of use and reduction of animal stress favors oral delivery of JNJ-40346527 (i.e., vs. added intra-dermal injections). Taken together, reducing Mon/Mac levels can significantly decrease skin inflammation suggesting that targeting Mon/Mac may be an effective means to prevent new inflammation and to, at least partially, treat existing disease. Confirmatory studies using other Ps like animal models are needed to further test the idea.

In conclusion, the current study confirmed that macrophages were upregulated in dermal and epidermal layers of human lesional Ps skin. In mice, activation of an important Ps pathogenic pathway, the IL-23 pathway, induced significant tissue levels of TNFα^+^ Mon/Mac. Mon/Mac cells were the predominant immune population in the inflamed skin highlighted by high levels of the Ly6C^hi^MHC II^hi^ subset. Pharmacological reduction of the Mon/Mac population ameliorated several inflammatory endpoints demonstrating a pathophysiological role for these cells and suggesting potential value in the treatment of Ps.

## Materials and Methods

More detailed materials and methods are available in the Supplementary Materials.

### Human skin

Punch biopsies (4 mm diameter) of psoriatic lesional and adjacent non-lesional skin were obtained from four Ps patients under an AbbVie Institutional Review Board-approved protocol. Informed consent was obtained and the study was performed in adherence with the Declaration of Helsinki Principles. Biopsies were placed in HypoThermosol FRS tissue transport solution (BioLife Solutions, Bothell, WA) for site-to-site transfer.

### Animal models

Female C57BL/6 mice were purchased from Charles River Labs (Portage, MA). All animal studies were conducted under a protocol approved by AbbVie’s Institutional Animal Care and Use Committee and in accordance with the relevant guidelines and regulations. All studies were conducted in a blinded manner.

IL-23 induced skin inflammation was achieved as described previously^18^. Briefly, 1 μg of recombinant IL-23 (AbbVie), or PBS + 0.1% BSA (Sham) was intradermally injected into the dorsal side of one ear once a day (morning) for four days starting on day 0. Ear thickness was measured daily and mice were euthanized on either day 2 or day 4 for tissue harvesting.

To deplete/reduce macrophages, JNJ-40346527, or vehicle (ASD (i.e. amorphous solid dispersion) formulation) was dosed orally at 100 mg•kg^−1^, once a day in either a prophylactic or treatment regime (Fig. 3 and Supplementary materials).

### Histology and Immunohistochemistry

Standard procedures were used for histology and immunohistochemistry as previously described^18^ and detailed in the supplemental materials. All routine H&E staining and IHC were conducted using ST5010 autostainer and BondRX immunostainer, respectively (both from Leica, Wetzlar, Germany). Standardized IHC protocol was used for anti-IBA-1 (Wako Chemicals, Richmond, VA) staining. Stained tissue slides were digitized with a P250 pathology slide scanner (Perkin Elmer, Waltham, MA) and analyzed by HALO software (Indica Labs, Corrales, NM).

For human and mouse skin samples, 4 µm microtome sections were collected on slides for H&E and IHC staining. An analysis technique was chosen to minimize the impact of variations in samples since total skin area as well as the proportion of epidermis to dermis can change as the disease progresses. Also, human biopsy collection techniques can result in “edge effects” in the dermis. Thus, for human biopsies, IBA1^+^ staining was normalized to the length of epidermis evaluated, and data are presented as area (µm^2^) divided by length (µm). For mouse ear samples, a standard 5 mm length was analyzed for each type of staining, and data are reported as area (mm^2^ or µm^2^) for both the H&E and IHC assessment.

### Tissue processing and Flow Cytometry

Ear skin preparation for flow cytometry analysis was conducted as previously described^18^ and in supplementary materials.

To determine cellular lineage, skin cell extracts were treated with standard flow cytometry surface staining procedures. For intracellular staining, skin cell extracts were incubated at 37 °C plus 5% CO_2_ for 3 hours in presence of 1X protein transporter inhibitor (eBioscience, Carlsbad, CA) prior to the standard surface and intracellular staining procedures (Supplementary Materials). Subsequently, cells were acquired using BD FACSAria III flow cytometer (BD Biosciences, San Jose, CA). The FCS files were analyzed using FlowJo 10.2 software (FlowJo LLC, Ashland, OR).

### Measurement of gene expression

Animal whole ears were homogenized and the expression of various genes was determined using QuantiGene multiplex kit (ThermoFisher Scientific, Waltham, MA). Probe sets used in the multiplex measurement are detailed in the supplementary materials. All procedures followed manufacturer’s instructions. Data was acquired using FlexMAP 3D cytometer (Luminex, Austin, TX). All data were normalized by GeoMean to two housekeeping genes, *Hprt* and *Gapdh*. Normalized data were than compared to the control group (i.e. vehicle treated Sham animals) and presented as fold changes.

### Statistics

Prism 7 (GraphPad Software, San Diego, CA) was used for all the statistical analyses. All studies have been repeated at least once and confirmed. Data were analyzed using either an unpaired T-test, or a one-way/two-way ANOVA followed by a Bonferroni’s multiple comparison post-hoc analysis if the ANOVA revealed significance. A *P*-value less than 0.05 was considered significant. Data are shown as mean ± SEM.

## Supplementary information

Supplementary information file

Supplemental figures

## References

[1] Gudjonsson JE, Elder JT (2007). Psoriasis: epidemiology. Clinics in dermatology.

[2] Nestle FO, Kaplan DH, Barker J (2009). Psoriasis. The New England journal of medicine.

[3] Greb JE (2016). Psoriasis. *Nature reviews*. Disease primers.

[4] Kim J, Krueger JG (2015). The immunopathogenesis of psoriasis. Dermatologic clinics.

[5] Marble DJ, Gordon KB, Nickoloff BJ (2007). Targeting TNFalpha rapidly reduces density of dendritic cells and macrophages in psoriatic plaques with restoration of epidermal keratinocyte differentiation. Journal of dermatological science.

[6] Fuentes-Duculan J (2010). A subpopulation of CD163-positive macrophages is classically activated in psoriasis. The Journal of investigative dermatology.

[7] Wang H (2006). Activated macrophages are essential in a murine model for T cell-mediated chronic psoriasiform skin inflammation. The Journal of clinical investigation.

[8] Stratis A (2006). Pathogenic role for skin macrophages in a mouse model of keratinocyte-induced psoriasis-like skin inflammation. The Journal of clinical investigation.

[9] Leite Dantas R (2016). Macrophage-mediated psoriasis can be suppressed by regulatory T lymphocytes. The Journal of pathology.

[10] Ward NL (2011). Depletion of antigen-presenting cells by clodronate liposomes reverses the psoriatic skin phenotype in KC-Tie2 mice. The British journal of dermatology.

[11] Nair RP (2009). Genome-wide scan reveals association of psoriasis with IL-23 and NF-kappaB pathways. Nature genetics.

[12] Cargill M (2007). A large-scale genetic association study confirms IL12B and leads to the identification of IL23R as psoriasis-risk genes. American journal of human genetics.

[13] Capon F (2007). Sequence variants in the genes for the interleukin-23 receptor (IL23R) and its ligand (IL12B) confer protection against psoriasis. Human genetics.

[14] Kulig P (2016). IL-12 protects from psoriasiform skin inflammation. Nature communications.

[15] Guenova E (2015). IL-4 abrogates T(H)17 cell-mediated inflammation by selective silencing of IL-23 in antigen-presenting cells. Proceedings of the National Academy of Sciences of the United States of America.

[16] Tonel G (2010). Cutting edge: A critical functional role for IL-23 in psoriasis. Journal of immunology.

[17] Puig L (2017). The role of IL 23 in the treatment of psoriasis. Expert review of clinical immunology.

[18] Gauld SB (2018). Mechanistic and pharmacological assessment of murine IL-23 mediated psoriasiform dermatitis; implications for drug discovery. Journal of Dermatological Science.

[19] Singh TP (2016). Monocyte-derived inflammatory Langerhans cells and dermal dendritic cells mediate psoriasis-like inflammation. Nature communications.

[20] Suarez-Farinas M (2013). Suppression of molecular inflammatory pathways by Toll-like receptor 7, 8, and 9 antagonists in a model of IL-23-induced skin inflammation. PloS one.

[21] Chan JR (2006). IL-23 stimulates epidermal hyperplasia via TNF and IL-20R2-dependent mechanisms with implications for psoriasis pathogenesis. The Journal of experimental medicine.

[22] Kohler C (2007). Allograft inflammatory factor-1/Ionized calcium-binding adapter molecule 1 is specifically expressed by most subpopulations of macrophages and spermatids in testis. Cell and tissue research.

[23] Utans U, Arceci RJ, Yamashita Y, Russell ME (1995). Cloning and characterization of allograft inflammatory factor-1: a novel macrophage factor identified in rat cardiac allografts with chronic rejection. The Journal of clinical investigation.

[24] Tamoutounour S (2013). Origins and functional specialization of macrophages and of conventional and monocyte-derived dendritic cells in mouse skin. Immunity.

[25] Malissen B, Tamoutounour S, Henri S (2014). The origins and functions of dendritic cells and macrophages in the skin. Nature reviews. Immunology.

[26] Rodero MP, Hodgson SS, Hollier B, Combadiere C, Khosrotehrani K (2013). Reduced Il17a expression distinguishes a Ly6c(lo)MHCII(hi) macrophage population promoting wound healing. The Journal of investigative dermatology.

[27] Zigmond E (2012). Ly6C hi monocytes in the inflamed colon give rise to proinflammatory effector cells and migratory antigen-presenting cells. Immunity.

[28] Guilliams M (2014). Dendritic cells, monocytes and macrophages: a unified nomenclature based on ontogeny. Nature reviews. Immunology.

[29] Geissmann F (2010). Development of monocytes, macrophages, and dendritic cells. Science.

[30] Genovese MC (2015). Results from a Phase IIA Parallel Group Study of JNJ-40346527, an Oral CSF-1R Inhibitor, in Patients with Active Rheumatoid Arthritis despite Disease-modifying Antirheumatic Drug Therapy. The Journal of rheumatology.

[31] George, D. M., Hoemann, M. & Loud, J. In *2017 Medicinal Chemistry Reviews* Vol. 52 (ed Joanne J. Bronson) Ch. 9, 165–178 (ACS Division of Medicinal Chemistry, 2017).

[32] Ma HL (2008). IL-22 is required for Th17 cell-mediated pathology in a mouse model of psoriasis-like skin inflammation. The Journal of clinical investigation.

[33] Thaci D (2015). Secukinumab is superior to ustekinumab in clearing skin of subjects with moderate to severe plaque psoriasis: CLEAR, a randomized controlled trial. Journal of the American Academy of Dermatology.

[34] Saurat JH (2008). Efficacy and safety results from the randomized controlled comparative study of adalimumab vs. methotrexate vs. placebo in patients with psoriasis (CHAMPION). The British journal of dermatology.

[35] Scholzen T, Gerdes J (2000). The Ki-67 protein: from the known and the unknown. Journal of cellular physiology.

[36] Cai Y (2011). Pivotal role of dermal IL-17-producing gammadelta T cells in skin inflammation. Immunity.

[37] Lowes MA (2008). Psoriasis vulgaris lesions contain discrete populations of Th1 and Th17 T cells. The Journal of investigative dermatology.

[38] Paukkonen K, Naukkarinen A, Horsmanheimo M (1992). The development of manifest psoriatic lesions is linked with the invasion of CD8+ T cells and CD11c+ macrophages into the epidermis. Archives of dermatological research.

[39] Bos JD (1989). Predominance of “memory” T cells (CD4+, CDw29+) over “naive” T cells (CD4+, CD45R+) in both normal and diseased human skin. Archives of dermatological research.

[40] van Rooijen, N. & Hendrikx, E. In *Liposomes: Methods and Protocols, Volume 1: Pharmaceutical* Nanocarriers (ed Volkmar Weissig) 189-203 (Humana Press, 2010).

