# Supplementary material for: Monocytes/Macrophages play a pathogenic role in IL-23 mediated psoriasis-like skin inflammation
Source: Sci Rep. 2019 Mar 29;9:5310. doi: 10.1038/s41598-019-41655-7 (PMC6441056; doi:10.1038/s41598-019-41655-7)
Supplement: Supplementary file 1 — Supplementary information file [file 41598_2019_41655_MOESM1_ESM.docx]

# Title:

Monocytes/Macrophages play a pathogenic role in IL-23 mediated psoriasis-like skin inflammation

Yibing Wang*^1^, Rebecca Edelmayer^1^, Joe Wetter^1^, Katherine Salte^1^, Donna Gauvin^1^, Laura Leys^1^, Stephanie Paulsboe^1^, Zhi Su^1^, Isaac Weinberg^1^, Marian Namovic^1^, Stephen B. Gauld^1^, Prisca Honore^1^, Victoria E. Scott^1^, Steve McGaraughty^1^

**1** Dermatology, AbbVie Inc., 1 North Waukegan Rd., North Chicago, IL 60064, USA

# Supplementary Materials and methods

## Animal model

Female C57BL/6 mice weighing 18-22 grams (Charles River Labs, Portage, MA) were housed in a pathogen free environment of an American Association for Accreditation of Laboratory Animal Care approved facility, eight per cage on aspen chip bedding (Sanichip, Montville, NJ) with individual ventilation and were kept in a room with an average temperature of 72 ^o^F on a 12 hour light-dark cycle. The mice had free access to 2014 Teklad global 14% protein rodent diets (Envigo, Madison, WI), and water. The animals were acclimated to laboratory conditions for at least 1 week before testing.

IL-23 induced skin inflammation was established by intradermally injecting 1 μg of recombinant murine IL-23 (generated by AbbVie) solution (total 20 μL in sterile PBS with 0.1% BSA) into the dorsal side of a single ear in an isoflurane (Abbott, Abbott Park, IL) anesthetized mouse with a 30 gauge needle. IL-23 was injected once a day for four days starting on day 0. Sterile PBS with 0.1% BSA was injected similarly to separate animals that were sham controls. Ear thickness was measured daily prior to IL-23 or sham injections using a digital caliper (model number 700-18-20; Mitutoyo Corporation, Aurora, Illinois).

To deplete/reduce macrophages, JNJ-40346527 (generated by AbbVie Inc.) or vehicle (formulated in ASD) was given orally at 100 mg•kg^-1^ once a day (Q.D.), in either a prophylactic or treatment regime, till the end of the study (day 4). For prophylactic, JNJ-40346527 was dosed two days prior to the first injection of IL-23 (day -2) in order to fully deplete Mon/Mac cells and for treatment dosing it was administered starting on day 2.

## Histology and Immunohistochemistry

Human biopsies were placed in 10% NBF within 2 to 4 hours of biopsy collection and then fixed ≥36 hours in this solution between two foam biopsy pads in a tissue cassette before being paraffin-embedded (FFPE) and processed using ASP300S (Leica, Wetzlar, Germany).

Mouse ear skin samples were harvested and fixed ≥36 hours in 10% NBF between two foam biopsy pads in a tissue cassette. A razor blade was used to split ear samples at midline from distal ear pinnae to proximal ear. Two additional cuts were made on either side of midline to create two 3 mm wide rectangular tissue samples for FFPE tissue processing with ASP300S.

For human and mouse skin samples, 4 µm microtome sections were collected on slides for Hematoxylin and eosin (H&E) and immunohistochemistry (IHC) staining. All routine H&E staining and IHC were conducted using a ST5010 autostainer (Leica, Wetzlar, Germany) or automated immunostainer BondRX (Leica, Wetzlar, Germany). A standardized IHC protocol was used for anti-IBA1 (clone 019-19741; Wako Chemicals, Richmond, VA) staining.

All stained tissue slides were digitized for image analysis using a P250 whole slide digital pathology slide scanner (Perkin Elmer, Waltham, MA) with 20X objective and extended focus scanning parameters. Data were further analyzed using HALO digital analysis software (HALO; Indica Labs, Corrales, NM). For human biopsies, IBA1^+^ staining was normalized to the length of epidermis evaluated, and data are presented as area (µm^2^) divided by length (µm). For mouse ear samples, a standard 5 mm length was analyzed for each type of staining, and data are reported as area (mm^2^ or µm^2^) for both the H&E and IHC assessment.

## Tissue processing and flow cytometry

Ear skin preparation for flow cytometry analysis was conducted as previously described ^18^. Briefly, whole skin ears were finely minced and digested with RPMI 1640 supplemented with 1% Penicillin-Streptomycin (Life Technologies, Grand Island, NY), 10 mM Hepes, 2 mg/mL Collagenase XI, 0.1 mg/mL DNase and 0.5 mg/mL Hyaluronidase (Sigma-Aldrich, St Louis, MO) by shaking at 37 °C for 45 minutes. The reaction was stopped by adding ice cold RPMI 1640 supplemented with 10% Fetal bovine serum. The mixture was then vigorously vortexed for 15 seconds and filtered through 40 μM strainer to obtain cells in suspension. The absolute cell number was acquired by counting the cells using NucleoCounter™ NC-200 (ChemoMetec, Denmark).

Final concentration of antibodies (μg/mL) was indicated unless the initial stock concentration of the reagent was not provided by the vendor. In the latter case, the dilution factor was indicated.

| Fluorochrome | Antigen | Antibody Clone | Vender | Staining concentration (μg/mL unless noted) |
| --- | --- | --- | --- | --- |
| FITC | CD45R/B220 | RA3-6B2 | BD Biosciences | 3.33 |
| PE | NK1.1 | PK136 | BD Biosciences | 2.0 |
| PE | RORγt | Q31-378 | BD Biosciences | 0.67 |
| PE-CF594 | Ly-6C | AL-21 | BD Biosciences | 3.33 |
| PerCP Cy5.5 | CD3ε | 145-2C11 | BD Biosciences | 2.0 |
| PerCP Cy5.5 | CD11b | M1/70 | BD Biosciences | 4.0 |
| PE-Cy7 | CD11c | HL3 | BD Biosciences | 3.33 |
| PE-Cy7 | TNFα | MP6-XT22 | BD Biosciences | 0.67 |
| Alexa Fluor 647 | MHCII(I-A/I-E) | M5/114.15.2 | BD Biosciences | 0.5 |
| Alexa Fluor 647 | Ki67 | B56 | BD Biosciences | 1:40 (dilution) |
| APC-H7 | CD4 | GK1.5 | BD Biosciences | 1.0 |
| APC-eFlu780 | CD3ε | eBio500A2 | eBiosciences | 2.0 |
| BV421 | CD64 | X54-5/7.1 | BioLegend | 3.33 |
| BV605 | CD45 | 30-F11 | BD Biosciences | 2.0 |

For the surface staining procedure, cells were first washed with 1X PBS, pH7.0, (Life Technologies, GrandIsland, NY) twice and then incubated with Fixable Viability Dye 510 (BD Biosciences, San Jose, CA) for 15 minutes at room temperature. Cells were then washed twice with 1X Stain Buffer /FBS (BD Biosciences, San Jose, CA) to remove residual viability dye followed by incubation with fluorochrome conjugated antibodies that recognize various cell surface antigens for 30 minutes on ice. Afterwards, cells were washed twice with 1X Stain Buffer/FBS and either acquired by flow cytometer or subjected to intracellular staining.

For intracellular staining, surface stained cells were prepared using Transcription Factor Buffer Set (BD Biosciences, San Jose, CA) according to the manufacturer’s instructions. In brief, cells were fixed and permeabilized for 45 minutes on ice, washed once and then incubated with fluorochrome conjugated antibodies that recognize various intracellular antigens for another 45 minutes on ice. Afterwards, cells were washed twice and applied to flow cytometer for data acquisition.

## Cellular lineage identification

The cellular populations from skin were identified using flow cytometry based on the expression of the surface antigens. The gating hierarchy and representative data plots are showed in Supplementary Fig. S2 online

To investigate the cytokine and intracellular protein expression of immune cells from skin, intracellular staining and flow cytometry based methodology was applied. The gating hierarchy to assess intracellular protein expression in immune cells and representative data plots are showed in Supplementary Fig. S3 online

## Measurement of gene expression

Probe sets used in the Quantigene multiplex analysis were designed by the vendor. Detailed information of the genes that were measured is listed below.

| **Accession Number** | **Symbol** | **Sequence length** | **Probe set region** |
| --- | --- | --- | --- |
| NM_013693 | Tnf | 1619 | 590-1163 |
| NM_010552 | Il17a | 1171 | 105-707 |
| NM_016971 | Il22 | 1088 | 189-797 |
| NM_008361 | Il1b | 1328 | 414-950 |
| NM_019728 | Defb4 | 458 | 89-372 |
| NM_001 001303 | Gapdh | 1254 | 735-1001 |
| NM_013556 | Hprt | 1349 | 23-637 |
