# Supplementary material for: Monocytes/Macrophages play a pathogenic role in IL-23 mediated psoriasis-like skin inflammation
Source: Sci Rep. 2019 Mar 29;9:5310. doi: 10.1038/s41598-019-41655-7 (PMC6441056; doi:10.1038/s41598-019-41655-7)
Supplement: Supplementary file 2 — Supplemental figures [file 41598_2019_41655_MOESM2_ESM.pdf]

TITLE:

Monocytes/Macrophages play a pathogenic role in IL-23 mediated psoriasis-like skin inflammation

AUTHORS:

Yibing Wang, Rebecca Edelmayer, Joe Wetter, Katherine Salte, Donna Gauvin, Laura Leys, Stephanie Paulsboe, Zhi Su, Isaac Weinberg, Marian Namovic, Stephen B. Gauld, Prisca Honore, Victoria E. Scott, Steve McGaraughty

Supplementary Figure 1
Ly6C^{hi}MHC II^{hi} macrophages increased over time compared to other CD64^{+} Mon/Mac subsets in IL-23 mediated skin inflammation

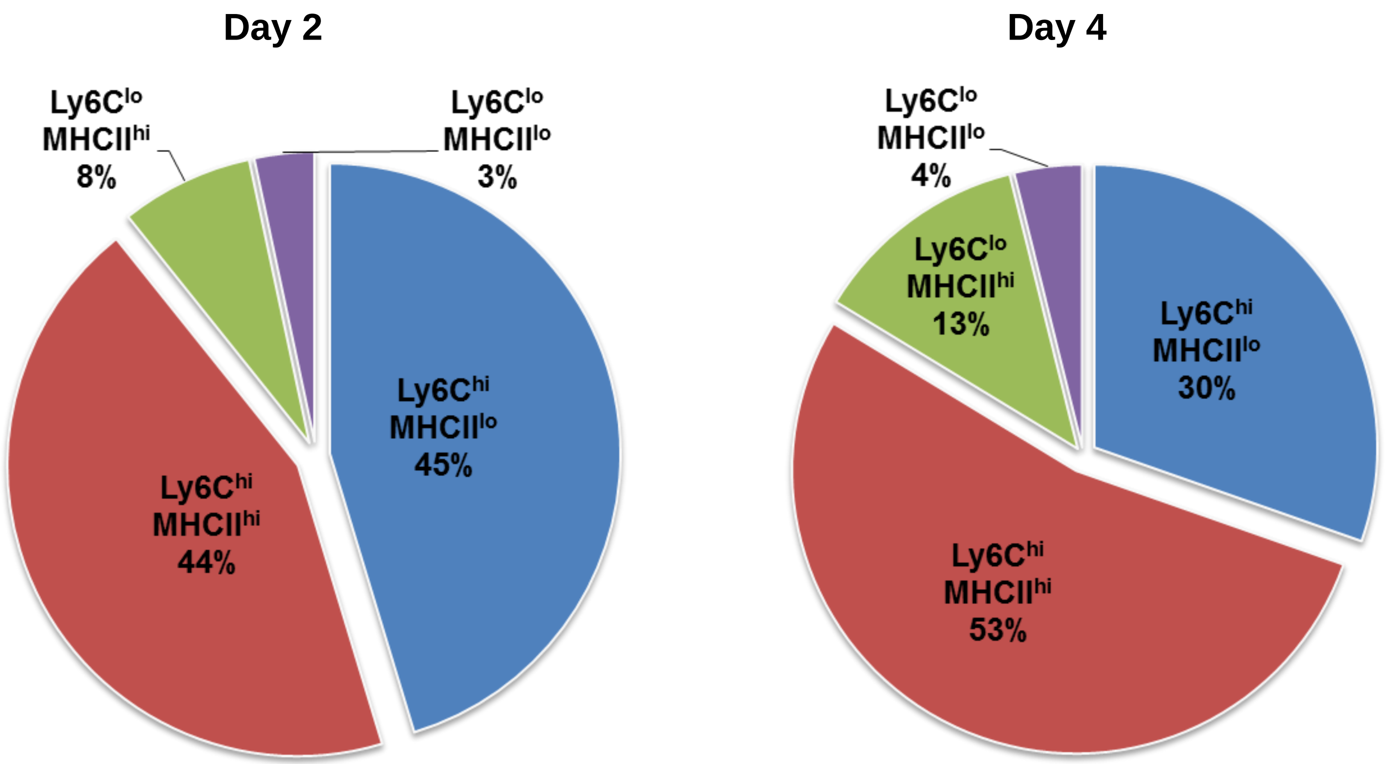

Supplementary Figure 2

Flow cytometry gating hierarchy for identifying Mon/Mac subpopulations by surface expression of lineage antigens

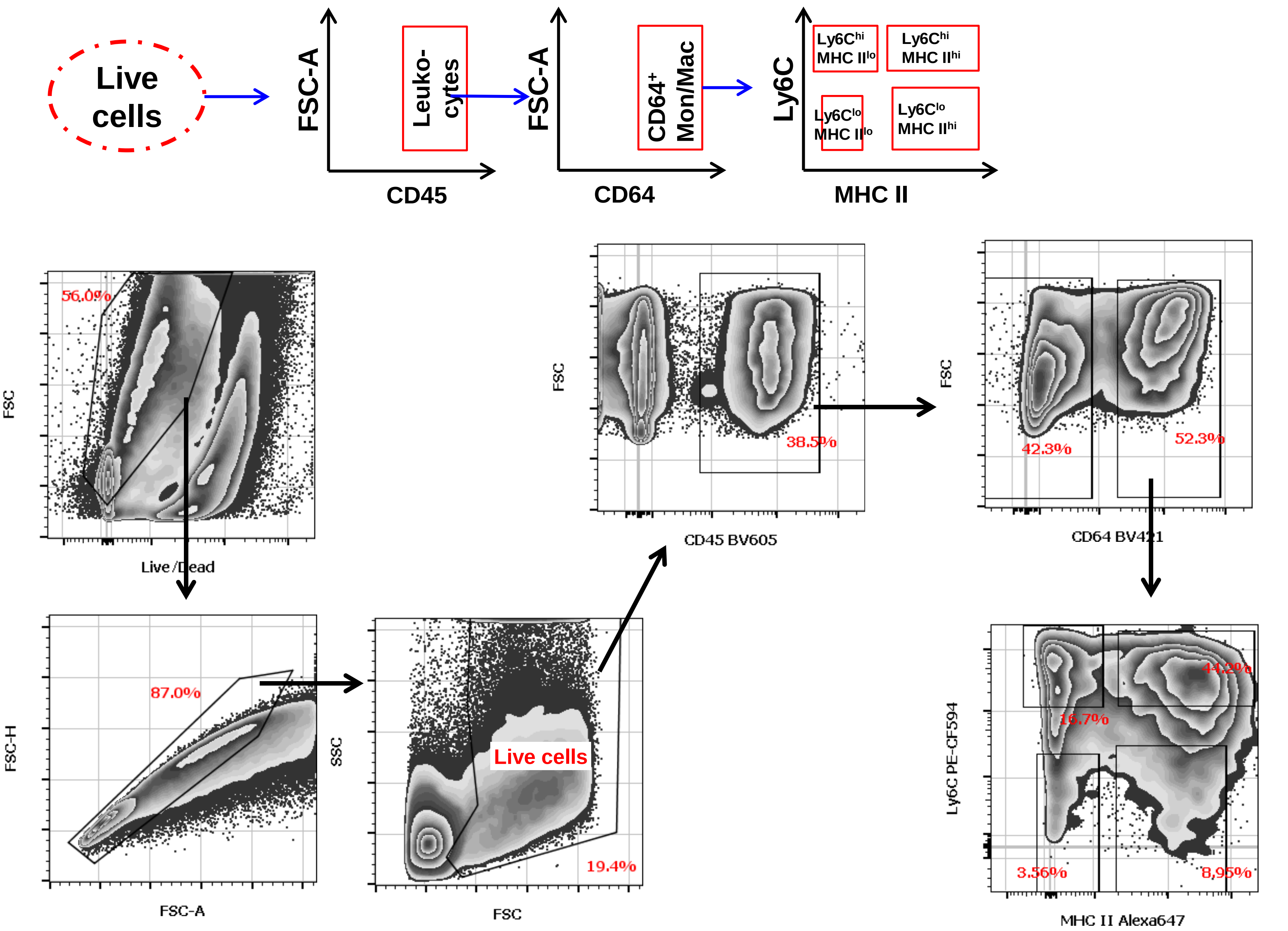

Supplementary Figure 3

Flow cytometry gating hierarchy of cellular populations for assessment of intracellular protein expression

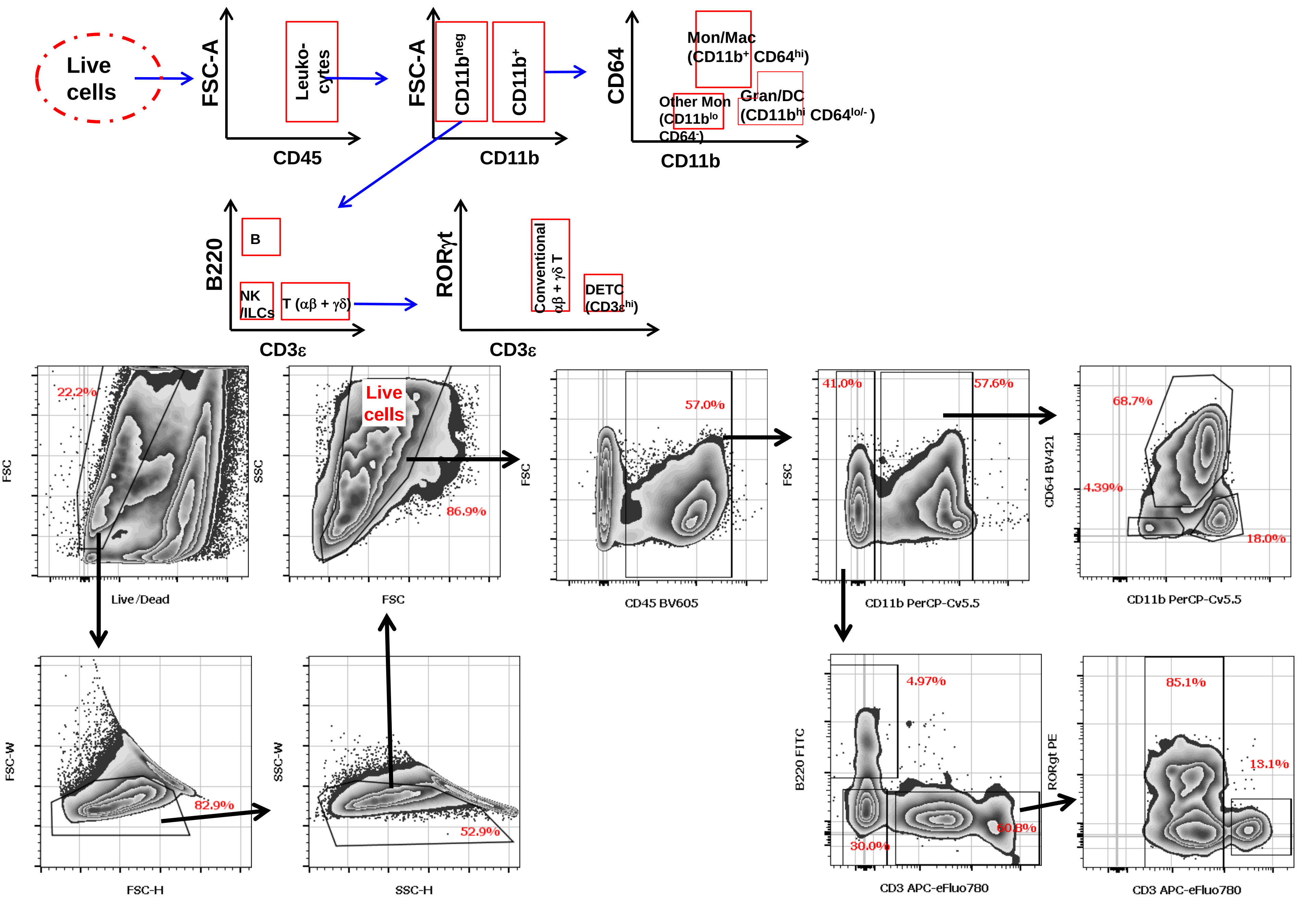

Supplementary Figure 4
TNFα and IL-17A expression in Granulocytes/Dendritic cell subsets were not elevated by IL-23 mediated skin inflammation

Gran/DC (neutrophils as majority)
(Live>$CD45^{+}$>$CD11b^{+}$>$CD11b^{hi}CD64^{lo/-}$)

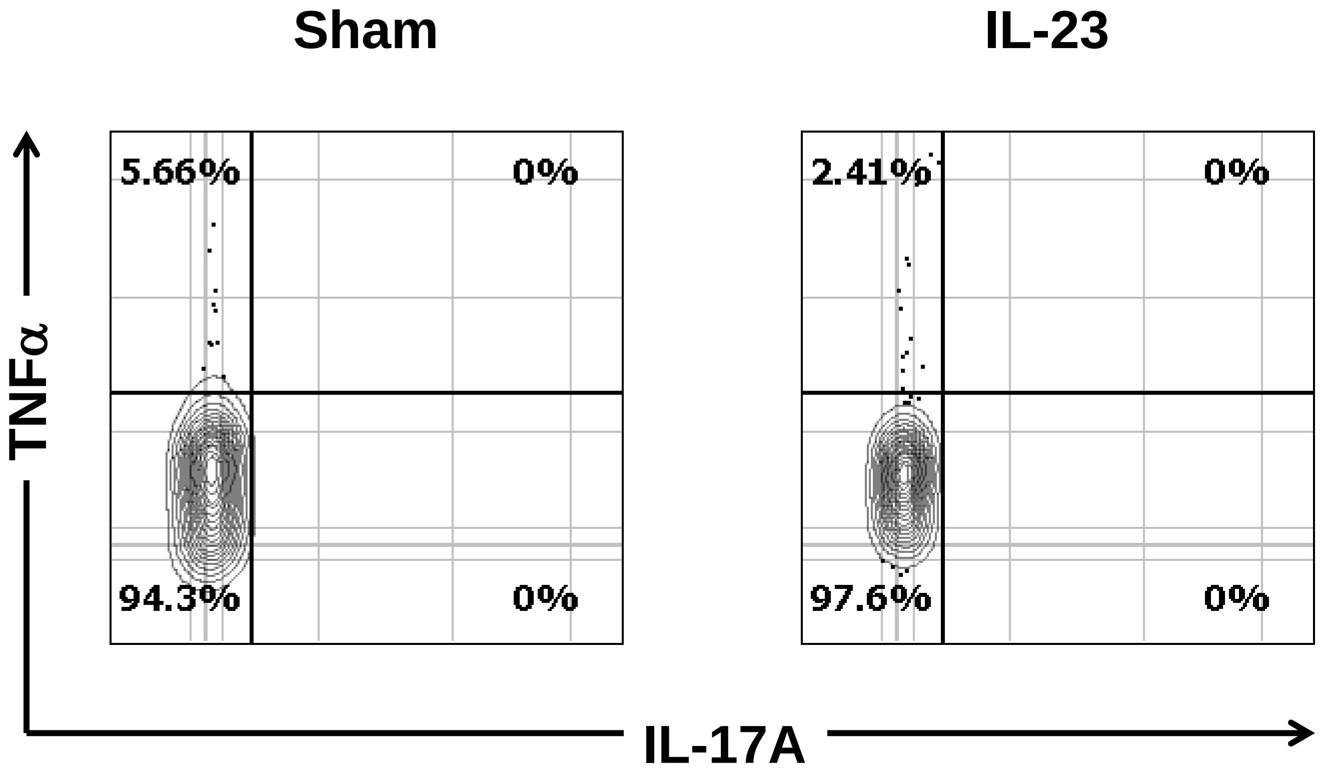

Supplementary Figure 5

IL-23 induced ear skin inflammation with increased accumulation of Mon/Mac and T cells

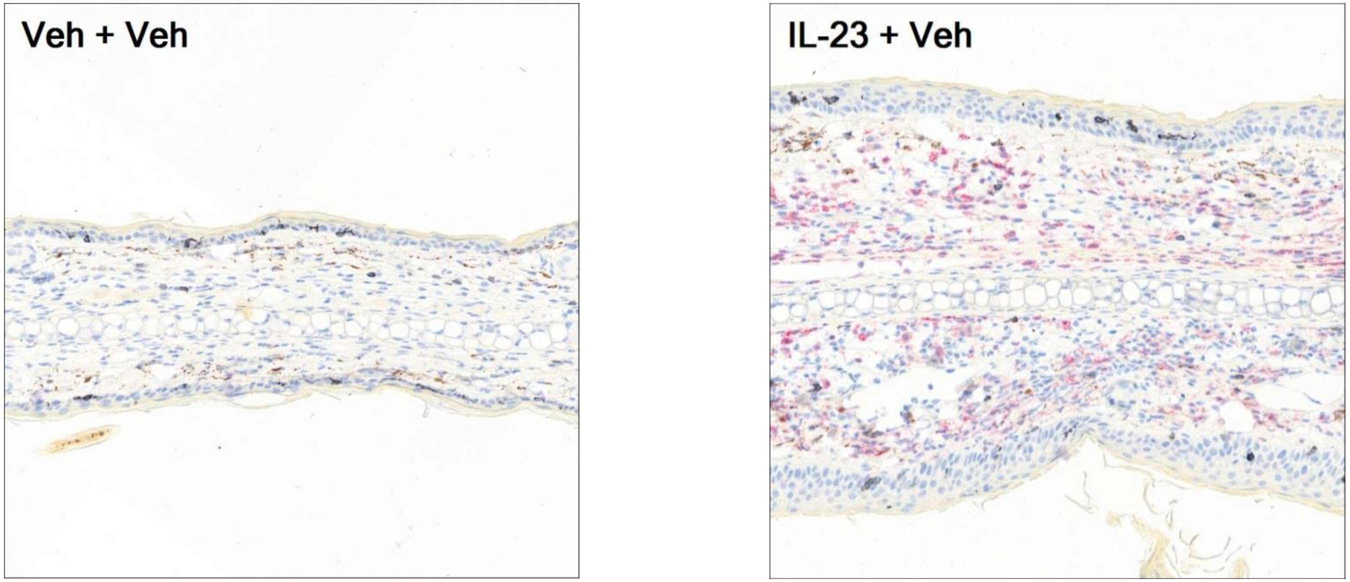

Red: IBA^{+}

Black: CD3^{+}
